# Defining a BMI Cut-Off Point for the Iranian Population: The Shiraz Heart Study

**DOI:** 10.1371/journal.pone.0160639

**Published:** 2016-08-10

**Authors:** Mohammad Ali Babai, Peyman Arasteh, Maryam Hadibarhaghtalab, Mohammad Mehdi Naghizadeh, Alireza Salehi, Alireza Askari, Reza Homayounfar

**Affiliations:** 1 Cardiovascular Research Center, Shiraz University of Medical Sciences, Shiraz, Iran; 2 Noncommunicable Diseases Research Center, Fasa University of Medical Sciences, Fasa, Iran; 3 MPH Department, Medical School, Shiraz University of Medical Sciences, Shiraz, Iran; 4 Research Center for Traditional Medicine and History of Medicine, Medical School, Shiraz University of Medical Sciences, Shiraz, Iran; 5 Health Policy Research Center, Shiraz University of Medical Sciences, Shiraz, Iran; TNO, NETHERLANDS

## Abstract

In this study we evaluated and redefined the optimum body mass index (BMI) cut-off point for the Iranian population based on metabolic syndrome (MeS) risk factors. We further evaluated BMI cut-off points with and without waist circumference (WC) as a cofactor of risk and compared the differences. This study is part of the largest surveillance programs conducted in Shiraz, Iran, termed the Shiraz Heart study. Our study sample included subjects between the ages of 20 to 65 years old. After excluding pregnant women, those with missing data and those with comorbid disease, a total of 12283 made up the study population. The participants underwent a series of tests and evaluations by trained professionals in accordance with WHO recommendations. Hypertension, abnormal fasting blood sugar (FBS), triglyceride (TG) and high density lipoprotein cholesterol (HDL) (in the context of the definition of metabolic syndrome) were prevalent among 32.4%, 27.6%, 42.1 and 44.2% of our participants, respectively. Women displayed higher rates of overall obesity compared to men (based on the definition by the WHO as higher than 30 kg/m^2^). Regarding MeS, 38.9% of our population had the all symptoms of MeS which was more prevalent among women (41.5% vs. 36%). When excluding WC in the definition of MeS, results showed that males tend to show a higher rate of metabolic risk factors (19.2% vs. 15.6%). Results of multivariate analysis showed that parallel to an increase in BMI, the odds ratio (OR) for acquiring each component of the metabolic syndrome increased (OR = 1.178; CI: 1.166–1.190). By excluding WC, the previous OR decreased (OR = 1.105; CI: 1.093–1.118). Receiver Operating Characteristic (ROC) curve analysis showed that the optimum BMI cut-off point for predicting metabolic syndrome was 26.1 kg/m^2^ and 26.2 kg/m^2^ [Accuracy (Acc) = 69% and 61%, respectively)] for males and females, respectively. The overall BMI cut-off for both sexes was 26.2 kg/m^2^ (Acc = 65%) with sensitivity and specificity of 69% and 62%, respectively. This cut-off had a positive predictive value of 54% and a negative predictive value of 76%. When we excluded waist circumference, the optimum BMI cut-off for acquiring metabolic risk factors in males decreased to 25.7 kg/m^2^ (Acc = 67%) and increased for women to 27.05 kg/m^2^ (Acc = 66%). Iranians are at higher risks of morbidity related to metabolic factors at a lower BMI cut-off and prompt action and preventive health policy are required to prevent and educate Iranians regarding diseases associated with obesity.

## Introduction

Many anthropometric indices have been used to identify the risk of morbidity and mortality associated with obesity. Among these indices body mass index (BMI) is most commonly used in large population, for identifying individuals at risk of cardiovascular disease, metabolic disease such as diabetes type 2 and insulin resistance and overall mortality [1].

The distribution of fat throughout the body, especially fat located in the abdominal area has been reported to have significant value in determining the risk of metabolic complications related to obesity [2]. Measures like waist circumference (WC) that correlate with abdominal obesity, independent from BMI, have been reported to be a great indicator for disease, especially coronary heart disease in obese patients [3–5]. A study evaluating the value of different anthropometric indices for the detection of fibrinogen and overall cardiovascular risk [6], found WC to be the best predictor of CVD risk and fibrinogen levels. This measure correlates closely to BMI and is considered in the definition of metabolic syndrome (MeS) as defined by the international diabetes federation [7]. MeS considers factors like hypertension and fasting blood sugar which are direct correlates of cardiovascular disease and diabetes, respectively. The condition also considers dyslipidemia which is also a risk factor for insulin resistance and itself is a risk factor for cardiovascular disease [8].

BMI cut-off points defined by the WHO, are based on the risk factors associated with development of disease, mostly cardiovascular disease. In light of the WHO expert consultation in 2004, it has become evident that a single BMI cut-off is unlikely to represent an equal accumulation of different risk factors for non-communicable disease among all ethnic groups and different populations worldwide [9–11].

Up to this date the optimum BMI for definition of disease has been a subject of great consideration among different researches. Studies have shown that Asians are likely to have a higher percent of adipose tissue, especially visceral adiposity, at lower BMI cut-off points than that reported by the WHO as standard cut-off points, which is based on studies in European and American populations [12, 13].

In Iran, research regarding obesity and its related risk factors has been scarce, although one recent study did show obesity rates to be increasing, especially in the male population [14]. This emphasizes the increasing need for a more precise cut-off of BMI as a predictor of disease. In this study we evaluated and redefined the optimum BMI cut-off point for the Iranian population based on metabolic syndrome risk factors. We also evaluated BMI cut-off points with and without WC as a cofactor of risk and compared the differences.

## Materials and Methods

### Study protocol and participants

This study is part of the largest surveillance programs conducted in Shiraz, Iran, termed the Shiraz Heart study. The goal of this screening program is to determine the risk factors associated with cardiovascular disease in the region. All those who participated in the research gave their written informed consent to participate in the study. The protocol of the research was approved by both the Ethics Committee and the Institutional Review Board of Shiraz University of Medical Sciences and was in accordance with the declaration of Helsinki.

Our study sample included subjects between the ages of 20 to 65 years old. After excluding pregnant women, those with missing data and those with comorbid disease, a total of 12283 made up the population of the study. A screening appointment was given to participants who agreed to enter the study and they were asked to fast for at least 10 hours prior to their blood tests. The participants underwent a series of tests and evaluations by trained professionals in accordance with WHO recommendations.

### Measurements

Anthropometric assessments were done while subjects had light cloths and were bare footed. WC was measured using an inelastic tape at midpoint of the inferior border to lowest ribs to the anterior superior iliac spine, after a normal expiration. The BMI was calculated as weight (Kg) divided by the squared height (m^2^). Height was measured to its nearest 0.1cm using a stadiometer (Seca 767, Japan) and weight was measured to its nearest 0.1 kg using a digital scale (Seca 767, Japan). Blood pressure (BP) was measured using a sphygmomanometer (Erka Perfect Aneroid, Germany). BP was measured 3 consecutive times with an interval of 5 minutes from the right arm, after each participant had rested for at least 10 minutes and finally a mean of the three measurements was considered as the blood pressure.

The biochemistry and blood tests including the fasting blood sugar (FBS), triglyceride (TG), cholesterol, high density lipoprotein cholesterol (HDL-c) and low density lipoprotein cholesterol (LDL-c) levels were assessed using venous blood samples obtained from each participant. For the assessment of fasting blood sugar (FBS) a glucose oxidize test was used (intra- and inter-assay coefficients of variation less than 2.1 and 2.6, respectively). The lipid profile was evaluated using an enzymatic approach (Parsazmoon Inc., Tehran, Iran). We used the Friedewald formula to calculate the LDL-c levels [12].

For the diagnosis of MeS we used the National Cholesterol Education Program’s Adult Treatment Panel (ATP) III revised guidelines as having three or more of the following criteria: a raised blood pressure (BP) defined as systolic BP > 130 mmHg and/or diastolic BP > 85 mmHg, TG ≥ 150 mg/dl, HDL-c < 40 mg/dl for male and < 50 mg/dl for females, FBS > 100 and WC > 88cm for women or WC > 102cm for men [15].

All the data

### Statistical analysis

Data was managed and analyzed using the Statistical Package for Social Sciences software, SPSS for windows, version 22 (SPSS Inc., Chicago, IL, USA). All the variables are displayed as means and standard deviations or percentage and frequency where appropriate. Our statistical inference was based on a 95% confidence interval (CI) and a significance threshold of 5%.

We used the logistic regression model to predict components of the MeS based on different BMI cut-off points. In the multivariate analysis we further adjusted BMI for sex and age.

To evaluate the appropriate BMI cut-off for the prediction of MeS, we used the Receiver Operating Characteristic (ROC) curve analysis. We used the presence of each of the criteria of the metabolic syndrome as the criterion and BMI as the continuous variable in the analysis. We reported the accuracy (Acc), sensitivity, specificity, positive predictive value (PPV), negative predictive value (NPV), positive likelihood ratio (PLR) and negative likelihood ratio (NLR) with and without considering WC, as one of the components of MeS for the definition of the appropriate BMI thresholds.

We obtained the optimal BMI cut-off using two different models. In the model referred to as convenience, cut-off was calculated using the Youden index [16]. In this model a point on the ROC curve is optimum which has the maximum sensitivity + specificity. We used this model for the definition of optimal BMI cut-off as it is the most commonly used model for defining cut-off points in medicine and has an easy calculation.

In the Second model [17] referred to as weighted, optimal cut-off is considered as the point on the ROC curve with minimum [(1 –sensitivity)^2^ + (1 –specificity)^2^] [17].

MeS was defined as having at least 3 of the 5 criteria defined for MeS [15]. So parsons with just one or two criteria were marked as non-MeS.

According to the high correlation between BMI and waist [7], we repeated our analysis once with excluding waist from the MeS criteria. In this analysis MeS was defined by having at least three of the four other criteria of MeS.

## Results

Baseline characteristics' of the study population is displayed in Table 1.

**Table 1:** Demographic characteristics of the study population.

Variables	Female (n = 6200)		Male (n = 6282)		Total (n = 12282)	
	Number	Percent	Number	Percent	Number	Percent
**Age groups**						
<30	551	8.9	305	5.0	856	7.0
30–39	1284	20.7	994	16.3	2278	18.5
40–49	2161	34.9	2429	39.9	4590	37.4
50–59	1684	27.2	1912	31.4	3596	29.3
*≥*60	520	8.4	442	7.3	962	7.8
BMI*						
<20	285	4.6	382	6.3	667	5.4
20–24.99	1786	28.8	2221	36.5	4007	32.6
25–29.99	2605	42.0	2664	43.8	5269	42.9
30–34.99	1196	19.3	678	11.1	1874	15.3
≥35	328	5.3	136	2.2	464	3.8
	**Mean**	**SD**	**Mean**	**SD**	**Mean**	**SD**
**Age**	44.95	10.38	46.36	9.25	45.65	9.86
**Waist**	94.63	12.40	93.58	10.56	94.11	11.54
**Systolic BP**	118.95	17.42	125.53	17.76	122.21	17.89
**Diastolic BP**	74.38	11.59	79.11	12.14	76.73	12.10
**Height**	158.30	6.51	171.84	7.06	165.01	9.59
**Weight**	68.07	11.97	76.45	13.05	72.23	13.20
**FBS**	97.57	30.96	99.16	29.18	98.35	30.10
**HDL**	49.34	11.73	44.48	10.51	46.93	11.40
**LDL**	105.35	27.75	106.70	27.48	106.02	27.62
**Cholesterol**	191.94	43.33	192.01	44.13	191.97	43.73
**TG**	143.46	82.44	169.57	102.27	156.37	93.68

BMI: Body mass index; FBS: Fasting blood sugar; HDL: High density lipoprotein; LDL: Low density lipoprotein: TG: Triglyceride

*Patients were first categorized based on their BMI by the definition of the WHO. According to this the majority of the Iranian population is overweight.

The men in our study had a similar frequency of overweight individuals when compared to the women in our study (42% and 43.8%, for males and females, respectively). However, women displayed higher rates of overall obesity compared to men (based on the definition of the WHO as higher than 30 kg/m^2^).

Hypertension, abnormal FBS, TG and HDL (in the context of the definition of MeS) were prevalent among 32.4%, 27.6%, 42.1 and 44.2% of our participants, respectively.

Males had higher rates of hypertension, abnormal FBS and TG (38.8% vs. 26.1%, 30.2% vs. 25.1% and 47.8% vs. 36.5%, respectively). Women had higher rates of abnormal HDL-c (54.4% vs. 33.9%) and higher rates of abnormal waist circumference (as higher than 102cm and 88cm for men and women, respectively) (89.2% vs. 50.6%). Overall among metabolic risk factors, abnormal HDL (44.2%), TG (42.1%) and hypertension (32.4%) were the most common risk factors.

Regarding MeS, 38.9% of our population had the all symptoms of MeS which was more prevalent among the women in our population (41.5% vs. 36%). When excluding waist circumference in the definition of metabolic syndrome, results showed that males tend to show a higher rate of metabolic risk factors (19.2% vs. 15.6%) (Table 2).

**Table 2:** Abnormal findings of the study population based on the definition of metabolic syndrome*.

Variables	Female		Male		Total	
	Number	Percent	Number	Percent	Number	Percent
**Abnormal Diastolic BP**	982	15.9	1600	26.3	2582	21.1
**Abnormal Systolic BP**	1294	20.9	1955	32.2	3249	26.5
**HTN¶**	1617	26.1	2355	38.8	3972	32.4
**Abnormal FBS**	1545	25.1	1822	30.2	3367	27.6
**Abnormal Waist**	5527	89.2	3071	50.6	8598	70.1
**Abnormal TG**	2257	36.5	2888	47.8	5145	42.1
**Abnormal HDL**	3341	54.4	2046	33.9	5387	44.2
**MeS**						
No	3565	58.5	3835	64.0	7400	61.2
Yes	2532	41.5	2159	36.0	4691	38.8
**MeS components–no.**						
No	224	3.7	853	14.2	1077	8.9
One	1302	21.4	1461	24.4	2763	22.9
Two	2039	33.4	1521	25.4	3560	29.4
Three	1608	26.4	1282	21.4	2890	23.9
Four	750	12.3	694	11.6	1444	11.9
Five	174	2.9	183	3.1	357	3.0
**MeS without waist**						
No	5151	84.4	4855	80.8	10006	82.6
Yes	950	15.6	1152	19.2	2102	17.4
**MeS without waist–no.**						
No	1224	20.1	1227	20.4	2451	20.2
One	2237	36.7	1925	32.0	4162	34.4
Two	1690	27.7	1703	28.4	3393	28.0
Three	774	12.7	908	15.1	1682	13.9
Four	176	2.9	244	4.1	420	3.5

BP: Blood pressure; HTN: Hypertention; FBS: Fasting blood sugar; HDL: High density lipoprotein; LDL: Low density lipoprotein: TG: Triglyceride; MeS: Metabolic syndrome

*Abnormality of the variables was defined based on the definition of the National Cholesterol Education Program’s Adult Treatment Panel III revised guidelines of MeS as: systolic BP >130 mmHg and/or diastolic BP> 85 mmHg, TG ≥ 150 mg/dL, HDL-c < 40 mg/dL for male and < 50 mg/dL for females, FBS> 100 and waist circumference of >88cm for women or >102cm for men.

¶Hypertension was defined as systolic Blood pressure>140 or diastolic blood pressure>90.

In our multivariate analysis, results showed that parallel to an increase in BMI, the odds ratio (OR) for acquiring each component of the MeS increased. Even when adjusted for age and sex the odds ratios remained significant (OR = 1.178; CI: 1.166–1.190). When we excluded waist circumference from the risk factors, the previous OR decreased (OR = 1.105; CI: 1.093–1.118) (Table 3).

**Table 3:** Logistic regression model for the prediction of metabolic syndrome based on BMI*.

	Odds ratio	95% confidence interval
**MeS**	1.190	1.178–1.202
**Adjusted for age and sex**	1.178	1.166–1190
**MeS components**		
One	1.277	1.246–1.309
Two	1.461	1.424–1.498
Three	1.576	1.536–1.617
Four	1.648	1.604–1.694
Five	1.694	1.640–1.749
**MeS without waist**	1.105	1.094–1.117
**Adjusted for age and sex**	1.105	1.093–1.118
**MeS components**		
One	1.065	1.051–1.079
Two	1.149	1.134–1.165
Three	1.197	1.178–1.216
Four	1.230	1.202–1.258

BMI: Body mass index; MeS: Metabolic syndrome

*The analysis was done both with and without considering waist circumference as a risk factor in the definition of BMI.

In the ROC curve analysis, the optimum BMI cut-off point for predicting metabolic syndrome was defined as 26.1 kg/m^2^ (Acc = 69%) and 26.2 kg/m^2^ (Acc = 61%) for males and females, respectively. The overall BMI cut-off for both sexes was 26.2 kg/m^2^ (Acc = 65%) with a sensitivity of 69% and a specificity of 62%. This cut-off had a PPV of 54% and a NPV of 76%.

**Fig 1:**
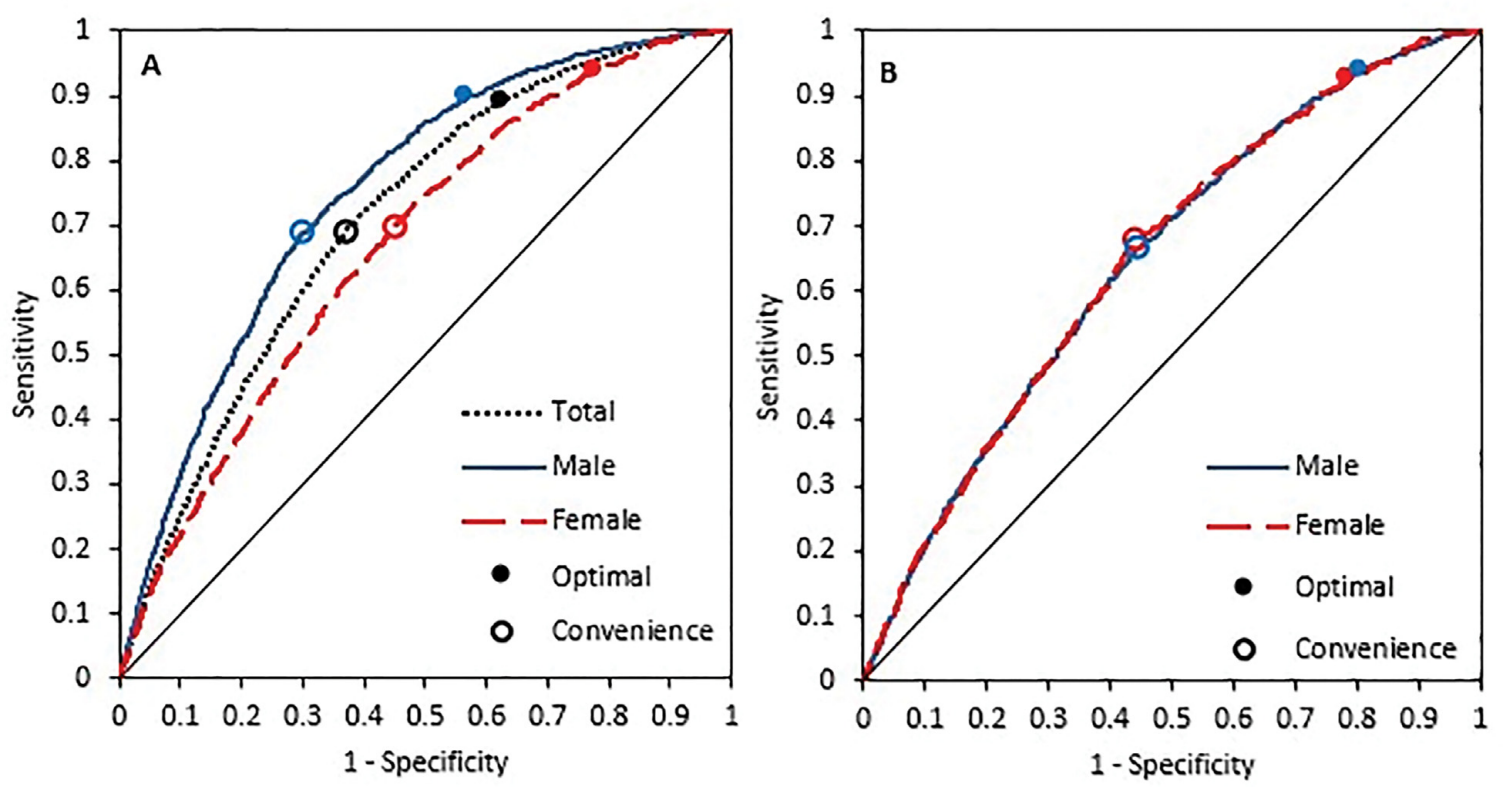
ROC curves for MeS using BMI. (A) In this chart metabolic syndrome was defined as having 3 of 5 components. (B) In this chart metabolic syndrome was defined without the waist component.

**Fig 2:**
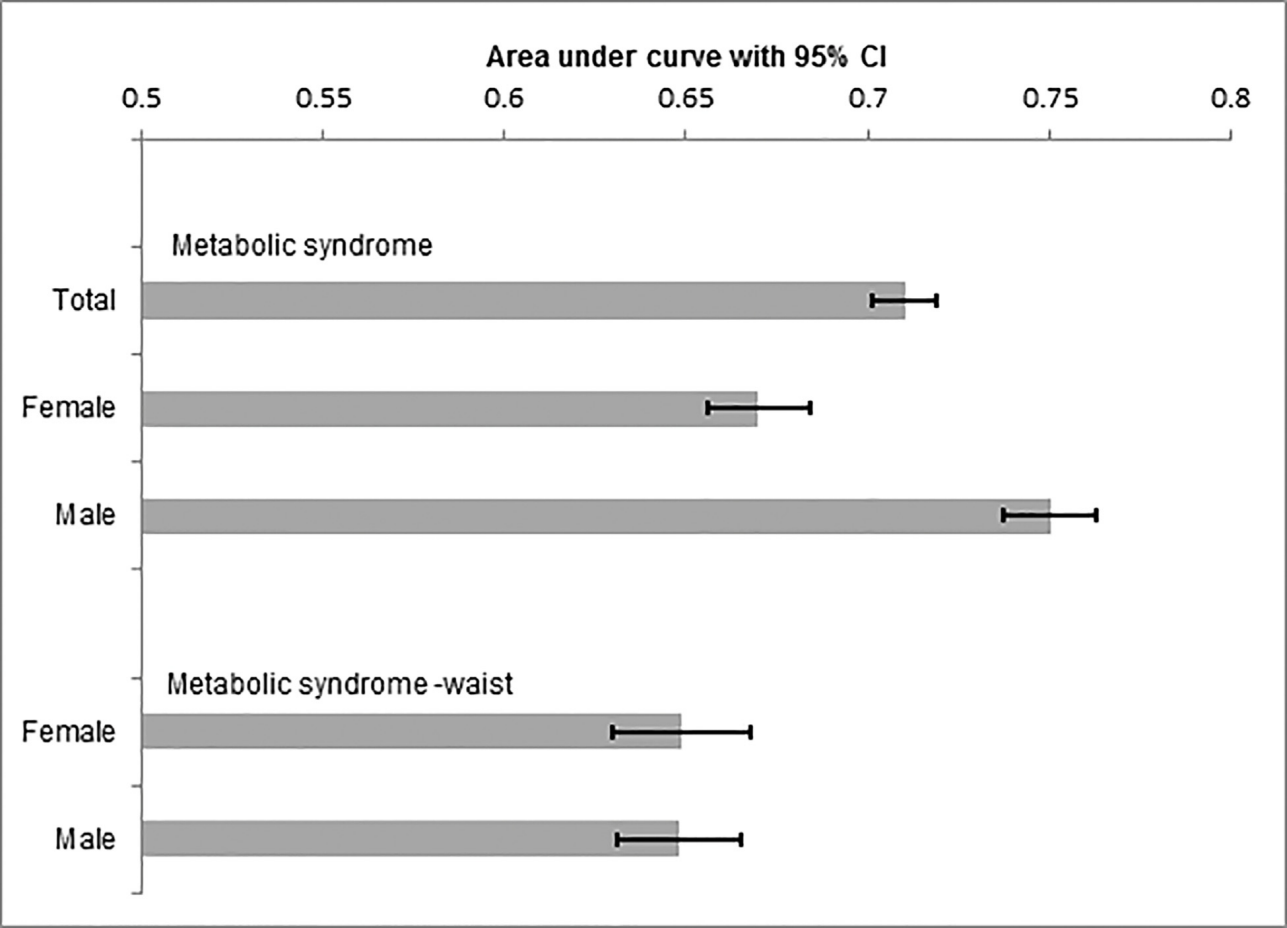
Area under curve with 95% confidence interval. In the three top bars MeS was defined with its five components. In the bottom bars MeS was defined with four components and waist was excluded from the MeS diagnostic criteria.

In the weighted model the optimum BMI cut-off for males and females was 23.9 kg/m^2^ (Acc = 60%) and 22.6 kg/m^2^ (Acc = 52%), respectively. This cut-off was 23.8 for both the sexes (sensitivity = 89.21 and specificity = 37.9). When we excluded waist circumference, the optimum BMI cut-off for acquiring metabolic risk factors in males decreased to 25.7 kg/m^2^ (Acc = 67%) and increased for women to 27.05 kg/m^2^ (Acc = 66%) (Table 4) (Figs 1 and 2).

**Table 4:** Receiver operating characteristic curve analysis for defining the ideal BMI cut-off point based on MeS components.

	Model*	Cut Point	Sensitivity	Specificity	PPV	NPV	Acc	PLR	NLR
**MeS**									
Total	Weighted	23.82	89.21	37.49	47.50	84.57	57.56	142.72	28.77
	Convenience	26.22	69.20	63.06	54.29	76.35	65.44	187.33	48.85
Female	Weighted	22.63	94.12	22.19	46.21	84.15	52.06	120.95	26.52
	Convenience	26.22	69.98	55.18	52.58	72.13	61.33	156.13	54.40
Male	Weighted	23.92	89.81	43.27	47.13	88.29	60.04	158.31	23.55
	Convenience	26.16	68.87	70.01	56.39	79.98	69.60	229.62	44.46
**MeS without waist**									
Female	Weighted	23.24	92.63	21.78	17.93	94.13	32.81	118.43	33.83
	Convenience	27.05	67.89	55.93	22.13	90.43	57.79	154.06	57.40
Male	Weighted	22.23	94.10	19.57	21.73	93.32	33.87	116.99	30.16
	Convenience	25.74	66.67	55.64	26.29	87.55	57.76	150.30	59.90

BMI: Body mass index; MeS: Metabolic syndrome; PPV: Positive predictive value; NPV: Negative predictive value; Acc: Accuracy; PLR: Positive likelihood ratio; NLR: Negative likelihood ratio

*Two models were defined for the calculation of BMI cut-off points. In the weighted model, optimum cut-off was defined as the point on the ROC curve with minimum [(1 –sensitivity)2 + (1 –specificity)2]. In the convenience model, optimum cut-off was defined as the point on the ROC curve with the maximum sensitivity + specificity.

## Discussion

Here we evaluated the appropriate BMI threshold based on the definition of metabolic syndrome in a large sample of the Iranian population. We found that Iranian men and women have the risk of developing metabolic syndrome at a BMI of 26.2, furthermore we also found that a BMI of 23.8 was the optimum point for screening purposes regarding metabolic syndrome. To the best of our knowledge this is the largest study in the Iranian population to evaluate the appropriate BMI cut-off point based on metabolic risk factors.

HE et al. [18] indicated that perhaps waist circumference and BMI should be assessed concomitantly for evaluating risk of CVD, since they both can have additional predictive values for CVD. Some studies have shown that metabolic syndrome may be associated with atherosclerotic change and type 2 diabetes even from childhood [19, 20]. Overall it seems that since MeS has WC as one of its components and BMI still remains the most commonly used index used in clinical practice, it would be advantageous to implement MeS in defining BMI cut-off points [1].

In the NHANES study in 2009 [21], Ervin reported a prevalence of 34% for MeS among the American population using the same definition of MeS as we used in our study, which was lower than that found in our study (34% vs. 38.8%). Considering the fact that the WC cut-off we used, is probably higher than what is specific for the Iranian population [22], the real prevalence rates of MeS is probably even higher than what we documented in our study.

Comparing men and women in our study, we found Iranian women to be more obese (BMI as higher than 30 kg/m^2^) than Iranian men and this was still the case even when we applied our own cut-off for BMI (26.2 kg/m^2^). We found metabolic risk factors to be more prevalent among women when considering WC as a component of MeS (41.5% vs. 36%), however when we excluded WC from the risk factors, we found them to be more prevalent among men (15.6% vs. 19.2%). One explanation for this could be that the WC cut-off we used in our study are significantly high for Iranian men (as compared to Iranian women) and perhaps a more specific WC cut-off should be applied.

In 2004, the WHO consultation group stated that based on the existing data, Asians may have higher chances of acquiring disease at a BMI cut-off once presumed as low risk for obesity related disease (lower than 25 kg/m^2^) and since then multiple studies have been conducted in the Asian region to evaluate the best threshold of BMI regarding risk of disease [9]. Majority of the studies point to the fact that Asians, especially those in south Asia, have a higher risk of developing metabolic and cardiovascular disease and have a higher percent of body fat, compared to their peer Caucasians living in the US and Europe with a similar BMI [23].

Al-Lawati et al. in 2008 [24], reported the optimum BMI cut-off for the Omani Arab population as 23.2 kg/m^2^ and 26.8 kg/m^2^ for men and women older than 20 years old, respectively. In another study [25] evaluating BMI based on metabolic risk factors conducted in Guatemala, as a developing country, they documented BMI cut-offs similar to our study as 24.7–26.1 kg/m^2^ for men and 26.5–27.6 kg/m^2^ for women.

In a sample of the Chinese population Dong and colleagues [26] evaluated 3006 individuals. They documented a cut-off of 25 kg/m^2^ for men and 24.5 kg/m^2^ for women as appropriate for the prediction of metabolic syndrome in the Chinese population.

For the Malaysian population, one study in 2009 [13], based on their definition of cardiovascular risk as hypertension, dyslipidemia and diabetes, reported a BMI cut-off of 23.5 and 24.9 kg/m^2^ for men and women, respectively.

In one study in the eastern province of Saudi Arabia [27] among a large population of adults over 30 years old, BMI cut-offs for detecting hypertension and diabetes were defined as 28.5 and 29.5 for men and 30.5 and 31.5 for women, although their ROC analysis did not show these cut-off points as having clinical value.

Wannamethee et al. [28] in a prospective study in 2010, found that a BMI of between 28–29 kg/m^2^ for men and a BMI of 29–30 kg/m^2^ was optimal for the diagnosis of diabetes in a large sample of residence within the UK.

Pan et al. [23] compared the accumulation of different risk factors including hypertension, diabetes, hypertriglyceridemia and hypercholesterolemia considering a similar positive predictive value between a Taiwanese population and a non-Hispanic Caucasian population from the US. They found that with a similar PPV, risk factors are much more prevalent in the Taiwanese population.

One of the confounding factors that influence the difference in cut-off points among studies, other than ethnic differences, could be the different age groups selected for the studies, as we know increased age can change body composition regarding total and distribution of body fat and especially metabolic factors [29].

When comparing our study results to other studies in the region and western countries, our findings indicates that the Iranian population, similar to those in other Asian countries, is at higher risks of acquiring metabolic diseases. As expected our cut-off points were lower than that stated by the WHO as the cut-off for morbidity (more than 30 kg/m^2^) [9]. One recent study by Zandieh et al. in Iran in 2012 [30], evaluated BMI cut-off points for metabolic risk factors among 3071 adults between the ages of 25–64 years old. Their cut-off points were similar to that documented in our study without the WC component (25.2 kg/m^2^ vs. 25.7 kg/m^2^ and 27.3 kg/m^2^ vs. 27.05 kg/m^2^ for men and women, respectively). When we considered WC in our definition of metabolic disease (MeS), we found that the cut-off point tends to increase for men, but decreases for Iranian women. This dramatic change may be because we considered the WC cut-offs defined by the National Cholesterol Education Program’s ATP III for the definition of MeS and perhaps a more population specific WC cut-off, would have yielded different results and prevented such a dramatic change amongst the male population. Based on one study in 2009 [22], the optimal WC for the Iranian population is 90cm for both men and women. We do not expect the result to have changed among the female population, since the cut-off points which we used in our study are very similar to that defined by the mentioned study as population specific (88cm vs. 90cm). The mentioned study defined the optimal WC for the Iranian population according to the risk factors of the metabolic syndrome. A prospective study published in 2009 by Hadeagh et al. [31] found that based on cardiovascular outcomes with a follow up of 7.6 years, the appropriate WC cut-off for the Iranian population based on the residence of the Tehran city, is 94.5cm for both sexes. Furthermore they found BMI cut-off points to be optimum at 26.95 for males and 29.19 for females.

This study was not without limitations. We studied the population referring from Shiraz city limiting our population sample to this geographical area which may have decreased the heterogeneity and the generalizability of the study results, however Shiraz is one of the most diverse cities in Iran regarding its ethnic groups and is considered the referral center for southern Iran, furthermore the large population size of the study may have relatively compensated this limitation. The cross sectional nature of our study does not allow a definite prediction of metabolic and cardiovascular outcome and long follow-ups in the context of prospective studies are needed. Since all of our participants were volunteers the ''Healthy Worker'' effect also remains a debatable issue in our study. In comparison to previous studies here we considered WC in our prediction of BMI cut-off points and as stated before WC independently correlate with cardiovascular disease and this is one of the strong points of our study.

Overall our study supported the idea that Asian countries require lower BMI cut-off points for predicting obesity related disease. Iranians are at higher risks of morbidity related to metabolic factors at a lower BMI cut-off with an estimated 38.8% of the population having MeS and prompt action and preventive health policy are required to prevent and educate Iranians regarding diseases associated with obesity.

## Supporting Information

S1 File Data set that contain all variables used for results. (TXT)
